# Etude de la repolarisation chez les sportifs de race noire

**DOI:** 10.11604/pamj.2019.33.114.14241

**Published:** 2019-06-14

**Authors:** Oussama Ben Rejeb, Hela Ghali, Yosra Messaoudi, Anissa Gharbi, Imen Bouhlel, Samia Ernez, Abdallah Mahdhaoui, Gouider Jeridi

**Affiliations:** 1 Université de Sousse, Faculté de Médecine de Sousse, 4000, Sousse, Tunisie; 2 Hopital Farhat Hached Sousse, Service de Cardiologie, Sousse, Tunisie; 3 Faculté de Médecine de Sousse, Laboratoire de Recherche LR14ES05 «Interactions du Système Cardio-pulmonaire», Sousse, Tunisie; 4 Hôpital Sahloul Sousse, Service de Prévention et Sécurité de Soins, Tunisie; 5 Hopital Ibn El Jazzar Kairouan, Service de Cardiologie, Sousse, Tunisie

**Keywords:** Repolarisation, athlètes, mort subite, Repolarization, athletes, sudden death

## Abstract

**Introduction:**

Il est considéré que la mort subite qui survient lors d'une activité sportive touche un patient ayant une cardiopathie ignorée. L'ECG du sportif de race noire a été peu étudié et des particularités liées à l'ethnie ont été évoquées. Notre travail a pour objectif d'étudier le profil épidémiologique et les particularités de la repolarisation du sportif de race noire.

**Méthodes:**

Une étude descriptive a été menée durant 8 mois de mars à octobre 2014, incluant des sportifs de race noire sélectionnés parmi tous les sportifs qui étaient suivi dans le centre sectoriel de la Médecine et des Sciences de sports de Sousse. Le recueil de données a été réalisé grâce à un questionnaire medical.

**Résultats:**

Trente-cinq (35) athlètes ont été colligés, avec une prédominance masculine (94,28%), ayant un âge moyen de 24,34 ans. Quatre athlètes avaient une hypertrophie ventriculaire gauche à l'échographie cardiaque. Il y'avait 8 athlètes avec un BAV de 1^er^ degré et 8 athlètes avaient un HVG électrique. Les modifications du segment ST étaient plus marquées au niveau des dérivations précordiales. Cinq athlètes (14,2%) avaient des ondes T négatives en V2 et V3, et il s'agissait des mêmes athlètes qui avaient le sus décalage du segment ST dans ces mêmes dérivations. Un aspect de repolarisation précoce a été constaté chez 3 athlètes. Tous ces cas étaient type notch.

**Conclusion:**

Les sportifs de race noire semblent avoir des modifications électriques assez spécifiques dont il importe de connaitre. Toutefois, notre échantillon n'est pas suffisamment important pour affirmer ces résultats. Une étude comparative avec des sportifs de race blanche serait très intéressante.

## Abstract (en)

**Introduction:**

Sudden death that occurs during sporting activity affects patients with ignored heart disease. Black athlete's ECG has been little studied and the features of this ethnic group have been discussed. This study aims to study the epidemiological profile and the peculiarities of repolarization of black athletes.

**Methods:**

We conducted a descriptive study of black athletes selected among all the athletes followed in the sectoral Center of Sports Science and Medicine in Sousse over a period of 8 months from March to October 2014. Data were collected using a medical questionnaire.

**Results:**

Data on 35 athletes were collected, with a male predominance (94,28%), with an average age of 24,34 years. Four athletes had left ventricular hypertrophy on cardiac ultrasound. There were 8 athletes with atrioventricular block degree I and 8 athletes with electrical type of left ventricular hypertrophy (LVH). ST segment changes were more marked at the level of precordial leads. Five athletes (14.2%) had inverted T waves in V2 and V3. These were the same athletes who ST-segment depression in these same leads. Early repolarization was found in 3 athletes. All these cases had notch signaling.

**Conclusion:**

Black athletes have quite specific electrical modifications which are important to know. However, our sample is not sufficiently large to certify these results. A comparative study of white athletes would be very interesting.

## Introduction

La mort subite est un syndrome défini par la survenue naturelle et inattendue, dans l'heure suivant le symptôme initial, d'un arrêt cardio-respiratoire chez une personne jusqu'alors considérée en bonne santé [1,2]. Les atteintes cardio-vasculaires représentent plus de 90% des causes incriminées dans la survenue de mort subite chez un sportif [3], avec une incidence, selon les études de 0,5 à 2,5 par an, pour 100000 athlètes âgés entre 12 et 35 ans, ce qui reste inacceptable [4-6]. Dans le cadre des causes cardiovasculaires, le mécanisme de la mort subite est dans la grande majorité des cas, un trouble du rythme ou plus rarement une anomalie de la conduction. Et dans ce contexte, Il est actuellement considéré que la mort subite qui survient lors d'une activité sportive touche un patient ayant une cardiopathie ignorée. L'intérêt de l'électrocardiogramme (ECG) dans le dépistage de certaines cardiopathies silencieuses a été démontré et fortement recommandé [7] avant la délivraison du certificat de non contre-indication au sport. Toutefois, la pratique régulière d'une activité physique peut s'accompagner de modifications électrocardiographiques, morphologiques et fonctionnelles du système cardiovasculaire regroupées sous le terme du syndrome de « cœur d'athlète ». Certains tracés ECG peuvent présenter des particularités qui sont difficiles à différencier d'authentiques phénomènes pathologiques. En effet, des troubles majeurs de la repolarisation à l’effort sont décrits chez 15% des athlètes [7]. Ces modifications secondaires à la pratique sportive varient avec la susceptibilité individuelle, l'intensité de l'entraînement physique, le type de sport pratiqué [8] et la race du sportif [9]. Le sportif de race noire a été peu étudié et son ECG pourrait présenter des particularités liées à l'activité physique. Partant de cette idée, nous nous sommes proposé à partir de ce travail d'étudier les particularités de la repolarisation chez des sportifs de race noire étiquetés indemnes de pathologie cardiovasculaire et ainsi établir un profil électro cardiographique dans une population de sportifs de race noire pouvant aider le médecin dans sa visite de non contre-indication à la pratique sportive.

## Méthodes

Il s'agit d'une étude descriptive rétrospective menée sur 8 mois, de mars à octobre 2014, incluant les sportifs professionnels de race noire sélectionnés parmi tous les sportifs qui étaient suivis régulièrement au centre sectoriel de la Médecine et des Sciences des sports de Sousse ou ceux qui ont été adressés au service de cardiologie de l'hôpital Farhat Hached de Sousse dans le cadre de la visite médicale de non contre-indication (VMNCI) à la pratique du sport et pour qui une aptitude au sport a été délivrée. Donc, le principal critère d'inclusion était les sportifs de race noire. Ont été exclus ceux chez qui on a découvert une cardiopathie structurelle ou une anomalie électrique qui a motivé leurs inaptitudes et ceux en apparence sains mais aux antécédents familiaux de mort inexpliquée ou de cardiopathie familiale. Le recueil des données à été réalisé grâce à un questionnaire médical contenant des données anamnestiques, biométriques (âge, sexe, taille, poids, indice de masse corporelle (IMC)), les données électro cardiographiques au repos et les données de l'échographie cardiaque trans thoracique.

**Figure 1:**
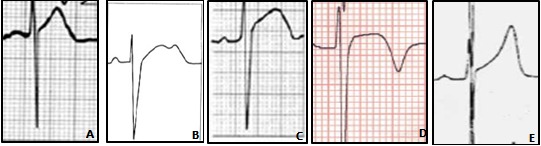
Répartition des atypies de la repolarisation selon la classification de Plas [16] A=type A (n=10; 28,5%); B=type B (n=1; 2,8%); C=type C (n=2; 3,6%); D=type D (n=5; 14,2%); E=type T (n=9;25%)

**Données de l'électrocardiogramme; définitions**: les électrocardiogrammes ont été consultés à partir des dossiers des sportifs: ces tracés ont été enregistrés au repos par un électrocardiographe selon les normes standards avec une vitesse de déroulement à 25mm/s et un gain de 10mm pour 1 milli Volt. Les différentes mesures ont été réalisées avec une réglette. Nous avons mesuré successivement les intervalles suivants: l'intervalle RR au niveau de la dérivation D2, l'intervalle PR (on l'a mesuré aussi en D2, entre le début de l'onde P et le début de QRS), la durée du QRS, l'indice de Sokolow, et une évaluation de la repolarisation [10,11]. Ainsi, l'étude de la repolarisation repose sur l'analyse de: segment ST: dans l'optique de chiffrer les modifications du segment ST, nous avons calculé la position du point J par rapport à la ligne isoélectrique. Le sus ou sous décalage de ST a été chiffré en mm; la morphologie de l'onde T; les ondes U: se sont de petites ondes positives qui surviennent juste après la fin des ondes T. Elles sont bien visibles dans les dérivations précordiales et correspondent habituellement à une repolarisation tardive; l'intervalle QT: pour minimiser le risque d'erreur, on a opté pour le calcul d'une moyenne à partir de 3 dérivations D2, V2 et V5: au niveau de chaque dérivation on a dessiné 2 lignes; la première est la ligne isoélectrique et la 2^ème^, ligne est la tangente de la partie descendante de l'onde T. Le QT est mesuré entre le début du QRS et l'intersection entre les 2 lignes. -QT mesuré= (QT en D2+QT en V2+QT en V5)/3 -QT Corrigé par la formule de Bazett=QT mesuré /√RR (en sec) [12]; la présence d'une encoche onde J au niveau de la partie terminale de QRS (Figure 1) [13-15]: ce sont plutôt des encoches (Notch) situées au niveau de la partie terminale des QRS. Elles font partie du syndrome de repolarisation précoce (dont l'aspect électrique est défini par l'élévation du point j>=1mm ou 0,1mv, représentée par un aspect terminal du QRS en notching (crochetage positif de la partie terminale du QRS) ou en slurring (cassure douce de la pente du QRS correspondant à la jonction avec le segment ST) dans au moins 2 dérivations contigües; les classes des atypies de la repolarisation chez le sportif selon Plas [16]: 1) la repolarisation de type T, plus nette en dérivations précordiales où la hauteur de l'onde T peut tripler; 2) la repolarisation de type A, caractérisée par le redressement de la partie initiale de ST qui devient oblique et ascendante. L'ensemble ST-T conserve cependant une positivité importante; 3) la repolarisation de type B comporte un segment ST également oblique ascendant avec une onde T bifide; 4) la repolarisation de type C est caractérisée par un segment ST large qui englobe complètement l'onde T; 5) la repolarisation de type D se présente comme un Sus-décalage de ST, curviligne, souvent moins marqué que dans les autres types, mais qui aboutit à une T inversée et pointue. Cet aspect est proche de celui observé en pathologie coronarienne d'ischémie-lésion.

La saisie et l'analyse des données ont été effectuées à l'aide du logiciel SPSS 11.5. Elle a comporté un calcul des fréquences absolues et relatives pour chacune des variables qualitatives, et un calcul des moyennes, des médianes et des écarts type pour les variables quantitatives. Pour tester statistiquement les croisements effectués entre certaines variables, nous avons utilisé, pour la comparaison de poucentage, le test de X^2^ ou Fisher quand les effectifs étaient insuffisants et le test t de Student, ANOVA ou U de Mann-Whitney pour la comparaison de moyennes.

## Résultats

**Description de la population d'étude**: la population étudiée a inclus 35 athlètes de race noire, avec une nette prédominance masculine (94,28%). L'âge moyen était de 24,34 ans avec des extrêmes entre 17 et 32 ans. Parmi nos athlètes de race noire, 4(11,4%) étaient de nationalité Tunisienne. Les autres sont des athlètes des pays de l'Afrique de l'Ouest provenant de plusieurs pays. Le sport pratiqué était majoritairement du football (94,28%). Aucun athlète n'a rapporté l'existence d'une maladie héréditaire dans la famille. Aucun signe fonctionnel n'a été rapporté, type dyspnée, douleurs thoraciques, palpitations ou lipothymies. Le poids moyen des athlètes a été de 75,45 kg±9,93 et la Taille moyenne a été de 173,74 cm ±6,6, avec un IMC moyen de 24,98 kg/m^2^ ±3,02. La PAS a été de 117, 71mmHg ±6,56; la PAD de 67,57 mm Hg±6,45.

**Données échographiques et de l'électrocardiogramme**: le nombre d'athlètes avec hypertrophie ventriculaire gauche (HVG) à l'échographie transthoracique (ETT) a été de 4(11,42%) chez qui le septum interventriculaire (SIV) variant de 11 à 13mm (Tableau 1). La fréquence cardiaque moyenne a été de 65 bpm (minimum =50bpm; maximum= 93 bpm). L'espace PR moyen a été calculé à 169,08ms ±34,61. Il y avait 8(22,9%) athlètes avec un bloc auriculo-ventriculaire de 1er degré. L'onde P avait une durée moyenne de 116 ms avec des extrêmes allant de 80 à 200ms. La durée moyenne du complexe QRS a été calculée à 83,05ms ±15,63. L'axe électrique du cœur a été normal chez tous les athlètes avec une moyenne de 58,85°. L'indice de Sokolow variait entre 18 et 43 mm avec une moyenne de 29,17 mm. Huit athlètes (22,9%) avaient une HVG électrique.

**Tableau 1:** Données de l’échographie transthoracique (ETT) chez les sportifs de race noire

	Minimum	Maximum	Moyenne	Déviation standard
SIV (mm)	7, 00	13, 60	10,9	1,49
PP (mm)	6,00	13,00	10,1	2,26
DTDVG (mm)	44,00	55,00	49,6	3,25
FEVG (%)	55,00	81,00	69,23	7,05
DTDVD (mm)	23,00	28,00	25,50	2,08
SOG (cm^2^)	9,00	22,00	13,25	5,96
PAPS (mm Hg)	15,00	34,00	21,33	7,36

**Etude de la repolarization**: le QT moyen mesuré a été de 397,31ms ±36,72 DS. Le QT calculé (QTC) selon la formule de Bazett a été de 416,88ms ±30,91 DS avec des valeurs extremes allant de 329 à 470ms. 5 athlètes (14%) avaient un QTC >440ms, ils étaient tous de sexe masculin.

**Étude du segment ST**: les modifications du segment ST étaient plus marquées au niveau des dérivations précordiales avec surtout un sus décalage de ST au niveau de V2 V3 V4. 10 (28,5%) athlètes ont présenté un sus décalage de ST (variant de 0,5 à 3 mm) dans les dérivations V2, V3, et 5 (14,2%) parmi ces athlètes avaient aussi un sus décalage en V4. Le sous décalage de ST a été constaté chez 2 athlètes (0,57%) et il a été confiné aux dérivations latérales (V5V6): ces 2 athlètes avaient en plus une HVG électrique et un aspect d'HVG dilatation à l'ETT (aspect en faveur d'un cœur d'athlète).

**Analyse de l'onde T**: les ondes T négatives étaient plus fréquentes au niveau des dérivations précordiales (surtout en V3 V4). 5 Athlètes (14,2%) avaient de façon simultanée des ondes T négatives en V2 et V3. Et il s'agissait des mêmes athlètes qui avaient le sus décalage de ST dans ces mêmes dérivations. Trois (3) athlètes (8,5%) avaient des ondes T négatives en latéral (précédées d'un sous décalage de ST chez 2 athlètes et isolée chez le 3^ème^ athlète) (Tableau 2).

**Tableau 2:** Aspect de l’onde T dans les différentes dérivations

Derivations	Positive	Negative	Plate	Diphasique
**D1**	29	0	6	0
**D2**	30	4	1	0
**D3**	28	7	0	0
**AVL**	15	0	20	0
**AVF**	28	6	3	0
**AVR**	4	31	0	0
**V1**	0	34	1	0
**V2**	30	5	0	0
**V3**	28	7	0	0
**V4**	28	7	0	0
**V5**	31	4	0	0
**V6**	32	3	0	0

**Étude simultanée des modifications du segment ST et de l'onde T chez les athlètes de race noire**: (Figure 1) les types de repolarisation les plus fréquents selon la classification de Plas ont été Le type A et le type T.

**Repolarisation précoce chez les athlètes de race noire**: un aspect de repolarisation précoce a été constaté chez 3 athlètes (2 localisations latérales et 1 localisation inférieure). Tous ces cas étaient type notch.

**Étude de l'onde U**: une onde U a été présente chez 12 athlètes (34,3%).

## Discussion

**Description de la population d'étude**: la mort subite d'un sportif est un syndrome défini par la survenue naturelle et inattendue d'un arrêt cardiorespiratoire chez une personne jusqu'alors considérée en bonne santé [1,2]. On distingue alors la mort subite du jeune sportif, âgé de moins de 35 ans et du sportif adulte de plus de 35 ans [17]. Les données épidémiologiques de la mort subite d'origine rythmique sont difficiles à recenser. En effet, d'une étude à l'autre les chiffres varient beaucoup en fonction des critères pris en compte: les causes, les symptômes, ou en fonction des variations génétiques d'un pays à l'autre. L'âge est de très loin le premier élément à prendre en considération vis-à-vis de l'incidence de la mort subite du sportif [18]. Ainsi, alors que le risque est de 0,5 pour 100 000 chez des adolescents, il s'élève à 6 pour 100 000 chez des adultes d'âge moyen [19,20]. Dans notre série, on a constaté que l'âge moyen était de 24,34 ans. Dans la plupart des séries qui ont étudié des populations d'âges différents, l'âge moyen est supérieur à 35 ans, atteignant parfois 45 ans. Aux États-Unis, dans le Minnesota, Maron *et al.* ont rapporté un risque de mort subite chez des jeunes athlètes de niveau universitaire de 0,5 pour 100 000, alors que Corrado *et al*. ont rapporté en Italie une incidence de 2,3 pour 100 000 [3, 21]. On constate aussi le fort déséquilibre entre les sexes, 95% des cas sont des hommes, contre seulement 5% de femmes [3], ce qui rejoint nos résultats. Le dimanche est le jour de la semaine le plus incriminé, jour où un grand nombre de sportifs s'adonnent à des exercices physiques; une forte prépondérance des cas de mort subite est observée l'été, le sport étant le plus fréquemment pratiqué à cette période de l'année [22].

**Cœur d'athlète: frontière entre physiologie et pathologie**: le terme cœur d'athlète regroupe l'ensemble des modifications cardio-vasculaires cliniques, électriques, fonctionnelles et morphologiques, qui peuvent être induites par la pratique d'un entraînement physique intense et prolongé [23]. Ce niveau est défini empiriquement et s'applique classiquement aux sportifs qui s'entraînent depuis plus de 6 mois au moins 6-8 heures par semaine, à une intensité supérieure au premier seuil ventilatoire (essoufflement marqué), soit au moins à 60-70% du VO2 max ou 70-80% de la fréquence cardiaque maximale individuelle [23]. Ceci veut dire qu'une pratique sportive modérée, y compris avec participation à des compétitions, ne modifie pas significativement l'ECG ni l'échocardiogramme du sportif. L'électrocardiogramme au repos du sportif de haut niveau d'entraînement peut être strictement normal 20% dans la série de Pelliccia *et al.* [24] ou montrer des atypies mineures (40% dans la même série). Parmi les particularités, les troubles de la repolarisation sont assez fréquents (11%) et surtout les plus préoccupants [25]. En effet, les anomalies de la repolarisation représentent la difficulté principale dans l'évaluation des ECG d'athlètes. D'après ses dernières recommandations sur l'interprétation de l'ECG d'athlète, l'ESC divise les atypies de repolarisation en 2 catégories: troubles de la repolarisation liés à la pratique sportive et ceux qui ne sont pas communs et donc nécessitant des investigations [26].

**Étude de la repolarisation chez les sportifs de race noire**: plusieurs travaux ont montré que la spécificité ethnique est une réalité chez les athlètes d'origine africaine avec une bradycardie moins marquée, des voltages QRS plus amples, des troubles de la repolarisation et une hypertrophie pariétale un peu plus prononcée. Les normes ont été déterminées chez les Caucasiens [27]. Pourtant, en ligue 1 de football en 2006, il y avait 186 joueurs d’origine étrangère. D’où l’importance d’adapter ces normes. La mort subite touche proportionnellement plus les Afro-Américains que les Caucasiens.

**L'intervalle QT**: il existe des désaccords à propos de l'influence de l'entrainement physique sur la durée de l'intervalle QT. L'athlète étant souvent bradycarde, ce qui induit un allongement notable de QT et nécessite pour l'interprétation une correction par la fréquence cardiaque (QTC) [28]. C'est la formule de Bazett qui est utilisée dans ce cadre. Compte tenu de cette bradycardie notoire chez le sportif, cette formule parait mal adaptée aux sportifs car elle surstime le QTC. Dans les séries de Van Ganse [29] ou de Palitini [30] ils ont constaté que le QTc est allongé dans les différentes dérivations en utilisant la formule de Bazett. Par contre Jordeans n'a pas démontré de différence significative chez les athlètes [31]. Dans ce cadre, plusieurs formules ont été développées pour calculer le QT chez les sportifs de haut niveau: 1) Bazett: QTcBa=QT.RR1/2; 2) Fridericia: [32] QTcFri=QT.RR1/3; 3) Framinghan: [33] QTcFra=QT+0.154.(1000-RR); 4) Hodges: [34] QTcHod=QT+105.(1/RR-1); 5) Linear: [35] QTcli=QT+K1.(1000-RR); 6) Exponential: QTcex=QT.RRK2.

Sara Young *et al.* [36] n'ont pas mis en évidence un QT plus long chez les athlètes et ce quel que soit la formule utilisée, et ont insisté que la formule la plus adaptée chez les sportifs avec une FC<60 bmn est la formule de Fridericia. La mesure de QT chez les sportifs y compris de race noire ne semble pas être différente de celle des sédentaires. Par conséquent l'allongement de QTc (> 500 ms avec une zone grise entre 460et 500 due à la formule utilisée) ne serait pas lié à la pratique sportive et inciterait à rechercher un syndrome de QT long congénital ou acquis(troubles électrolytiques, abus de drogues [37]). La valeur seuil à utiliser est 440 ms chez l'homme et 460 ms chez la femme [38]. Dans notre série, 5 athlètes avaient un QTc selon la formule de Bazett > 440 ms, mais tous au-dessous du seuil de 500 ms. Pour ces mêmes athlètes, le QTc en utilisant la formule de Fridericia est inférieur à 440 ms. Donc, en pratique, on recommande le calcul de QT selon la Formule de Bazett et une correction selon la formule de Fridericia chaque fois que le QT bazett est dans la zone grise (entre 440 et 500 ms). Dans un travail fait à la faculté de médecine de Tunis [39] comparant l'aspect de l' ECG entre 46 sportifs de race noire de l'Afrique de l'Ouest et 46 sportifs de race blanche, il n'y avait pas de différence statistiquement significative dans les valeurs de QT entre les 2 groupes (390 ms versus 405 ms chez les sportifs de race noire; p=0,07). C'est pour conclure que très probablement les sportifs de race noire n'ont pas de particularités relatives à l'intervalle QT en comparaison avec les sportifs de race blanche.

**Les ondes T**: les ondes T positives et amples représentent l'anomalie la plus fréquente chez le sujet sportif aussi bien de race noire que de race blanche [40]. Il a été constaté à travers plusieurs études que les athlètes de race noire présentent plus d'ondes T négatives que les athlètes de race blanche (22,8% contre seulement 3,7% avec p<0,001) [41]. Et lorsque présentes, ces ondes T sont surtout localisées au niveau des dérivations précordiales de V1 à V4 (dans 12,7%) et seulement 4,1% ont des ondes T négatives localisées au niveau des dérivations latérales. Ces résultats sont concordants avec nos résultats puisque 22,7% de nos athlètes avaient des ondes T négatives dont 14,2% en localisation précordiale droite. Papadakis *et al.* [42] dans leur travail qui a comparé les troubles de la repolarisation chez des athlètes de race noire et des sédentaires de race noire atteints de cardiomyopathie hypertrophiante (CMH) ont constaté une distribution des ondes T négatives au niveau latéral dans 77% contre seulement 3,8% au niveau des dérivations V1-V4 chez les sujets atteints de CMH. Dans ce même travail et au cours d'un suivi (69,7 ± 29,6 mois) des athlètes de race noire qui ont des ondes T négatives, 3 cas de CMH ont été diagnostiqués dont 1 a eu une mort subite récupérée. Les 2 cas avaient des ondes T négatives en latéral et en inférieur. Aucune cardiomyopathie n'a été diagnostiquée chez tous ceux avec une inversion des ondes T de V1 à V4. Ce qui vient consolider l'idée que des ondes T négatives de V1 à V4 est une variation ethnique du cœur d'athlète. Le Tableau 3 résume les résultats des principales études qui se sont intéressées à l'étude des ondes T chez les athlètes de race noire.

**Tableau 3:** Prévalence des ondes T négatives chez les sportifs de race noire et blanche selon différentes études

Etudes	Lieu	Nombre	Sexe	Race Noire	Race Blanche
		Noir/blanc		Atypies ECG T <0	Atypies ECG T<0
**Kervio *et al.* [****36****]**	France and Japan	96/118	M	21%/6,2%	6%/0%
**Papadakis *et al.* [****42****]**	France et UK	904/1819	M	Np/22,8%	Np/3,7%
**BALADY *et al.* [****44****]**	USA	97: race noire 192: race blanche	M	Np/23,7%	Np/8,3%
**Choo *et al.* [****45****]**	USA	835/408	M	Np/20,1%	Np/9,4%
**Magalski *et al.* [****46****]**	USA	1321/598	M	30%/2,6%	13%/0,2%
**Rawlins *et al.* [****47****]**	France et UK	240/200	F	Np/14%	Np/2%
**Zaidi *et al.* [****48****]**	UK	300/375	M	Np/21,7%	Np/5,1%
**Wilson *et al.* [****49****]**	QATAR	300/120	M	18/15,9%	5,8%/0%
**Riding *et al.* [****50****]**	QATAR	410/160	M	20%/13%	6,9%/1%

NP= non precise; ECG= Electrocardiogramme

**Le segment ST**: le sous-décalage doit faire toujours amener à rechercher une cardiopathie sous-jacente; 1) le sus-décalage du point J dans les dérivations précordiales (V1 à V3) ou périphériques (DII-DIII -aVF) peut accompagner une pratique sportive intense (repolarisation précoce) qui est plus fréquente chez les sportifs de race noire. 2) Le sus décalage du segment ST a été surtout rencontré de V1 à V3. C'est la modification électrocardiographique la plus fréquente. Si elle est classique chez le sujet sportif, cette anomalie pose des problèmes de signification lorsqu'elle s'associe à un bloc de branche droite (aspect évoquant un syndrome de Brugada).

**L'onde U**: l'onde U est généralement physiologique si son amplitude est modeste (inférieure à l'onde T ou inférieure à 0,3 mV) et s'il y a retour à la ligne isoélectrique entre T et U. Elle peut être vue dans toutes les dérivations, mais elle est généralement mieux visible en V2-V3, en particulier si la fréquence cardiaque est peu élevée [43]. Elle est présente dans 90% des cas en cas de fréquence cardiaque <65 bpm. L'onde U chez l'athlète de race noire est souvent bénigne mais elle peut gêner la mesure de l'intervalle Q-T si elle masque la fin réelle de l'onde T.

**Signification des différentes atypies de la repolarisation chez le sportif de race noire**: plas [16] insiste sur la nécessité de percevoir ces anomalies de la repolarisation comme un film électro cardiographique se déroulant au cours de la pratique sportive. Leur progression se fait toujours du type A vers le type D. Leur régression s'effectue spontanément à la fin des compétitions et s'opère progressivement, dans l'ordre inverse, sur une période d'au moins deux mois. Le type T serait le plus souhaitable pour un athlète de haut niveau .Il est assez souvent observé quand la condition physique est bonne. Le type D ayant une signification péjorative, traduisant un surentraînement cardiaque. Le type D n'a pas de signification coronarienne ce que confirme sa disparition à l'effort et l'origine de ces modifications serait métabolique.

**Limites de notre étude**: notre travail est la première étude clinique dans la région de Sousse ayant étudié la repolarisation chez des sportifs de race noire. Mais, il présente certaines limites: 1) la relative petite taille de l'échantillon; 2) une comparaison avec des athlètes de race blanche serait très intéressante; 3) l'impact pronostic est à déterminer, un suivi à moyen et à long terme est nécessaire.

**Considérations éthiques**: cette étude n'a pas posé de problème éthique puisqu' elle est rétrospective basée sur une consultation des dossiers médicaux après avoir un consentement oral des participants.

## Conclusion

Les atypies de la repolarisation à l'ECG du sportif de haut niveau restent une préoccupation majeure du médecin en particulier lorsqu'il s'agit de délivrer un certificat de non contre-indication à la pratique sportive. Ce travail vient apporter un argument de plus que le cœur d’athlète a ses raisons ethniques que les normes ne doivent pas ignorer: les sportifs de race noire semblent avoir des modifications électriques assez spécifiques dont il importe de connaitre. Ces données sont toujours un sujet de débat notamment en ce qui concerne la réalisation d'autres examens complémentaires devant un tel aspect à l'ECG. La réponse vient par Dr Carré au CHU de Rennes qui affirme que [23]: « La spécificité ethnique reste un diagnostic d’élimination qui ne doit pas nous empêcher de faire des examens ». Cependant, notre échantillon n'est pas suffisamment important pour affirmer ces résultats. Une étude comparative avec des sportifs de race blanche serait très intéressante. D'autre part, un suivi longitudinal sur plusieurs années de ces athlètes avec atypies de la repolarisation pourrait aider à une meilleure confirmation du caractère bénin de certains aspects ECG.

### Etat des connaissances actuelles sur le sujet

- Les atteintes cardio-vasculaires représentent plus de 90% des causes incriminées dans la survenue de mort subite chez un sportif;
- Le sportif de race noire a été peu étudié et son ECG pourrait présenter des particularités liées à l'activité physique.

### Contribution de notre étude à la connaissance

- Des données sur les caractéristiques éléctrocardiographiques des sportifs de haut niveau de race noire;
- Les athlètes de race noire semblent avoir des modifications électriques assez spécifiques dont il importe de connaitre. Ils ont tendance à avoir plus de troubles de la repolarisation en particulier plus d'ondes T négatives;
- Une étude comparative avec des sportifs de race blanche serait très intéressante.

## Conflits d’intérêts

Les auteurs ne déclarent aucun conflit d'intérêts.
